# Characterization of transcripts emanating from enhancer *Eβ* of the murine *TCRβ* locus

**DOI:** 10.1002/2211-5463.13079

**Published:** 2021-03-16

**Authors:** Faizan Uddin, Madhulika Srivastava

**Affiliations:** ^1^ Epigenetics Research Laboratory National Institute of Immunology New Delhi India

**Keywords:** bidirectional transcripts, enhancer RNA, eRNA, LncRNA, noncoding transcription, overlapping transcription

## Abstract

Enhancers are well established as critical regulators of gene expression, but the mechanisms underlying the molecular basis of their specificity and activity are only partly understood. One of the most exciting recent observations is the discovery of enhancer RNA (eRNA), a class of noncoding RNAs derived from enhancer regions. Transcription of developmentally regulated enhancers has been observed to be associated with their active state. The nature of transcripts (eRNA) and their functional attributes are diverse and context dependent. The majority of eRNA are nonpolyadenylated and present in low abundance owing to their low stability, and may represent transcriptional noise. However, some eRNAs have been reported to be reasonably long and stable, are enriched in nuclei, exhibit tissue‐specific expression and may contribute to enhancer function. Transcription of enhancers has been postulated to mediate enhancer function through either the act of transcription or via the transcribed RNA *per se* and is a useful feature to be analysed to understand mechanisms underlying enhancer activity. Enhancer *Eβ* at the murine *TCRβ* locus has been reported to exhibit enhanced occupancy of RNA polymerase II in developing thymocytes. Here, we investigated the transcriptional potential of *Eβ* in developing thymocytes and detected overlapping bidirectional transcripts at *Eβ* ranging between 0.7 and 1.7 kb. These noncoding transcripts are capped, polyadenylated, nuclear and expressed specifically in thymocytes. Delineation of these characteristics is important to further investigate their functional roles in mediating enhancer activity.

## Graphical abstract

Several enhancers in their active state exhibit enrichment of H3K4‐me3 and H3K27‐ac, accumulate RNA polymerase II and undergo unidirectional or bidirectional transcription. In this study, enhancer *Eβ* of the *TCRβ* locus was found to produce noncoding, convergent and bidirectional transcripts, consistent with its critical regulatory role, in developing thymocytes. It will be interesting to evaluate the functional and mechanistic significance of transcription at enhancer *Eβ*.

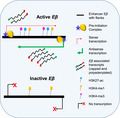

**Abbreviations**
3C: chromosome conformation capture
4C: circular chromosome conformation capture
DN: double negative
DP: double positive
eRNA: enhancer RNA
gDNA: genomic DNA
GSP: gene‐specific primer
lncRNA: long noncoding RNA
qPCR: quantitative PCR
RACE: rapid amplification of cDNA ends
RC: recombination center
RNAPII: RNA polymerase II
RT‐qPCR: reverse transcription quantitative PCR
ser‐5P: serine 5‐phosphorylated
SP: single positive
TCRβ: *T‐cell receptor β*
*TES*: *transcription end site*
TFs: transcription factors
TSS: transcription start site

Genesis of complex and multicellular life forms requires concerted activity of thousands of genes whose expression is precisely regulated during development [1]. The spatio‐temporal regulation of genes relies upon convoluted network of dynamic interactions between myriad nuclear components. These include DNA regulatory elements such as enhancers, promoters and insulators, proteinaceous components like transcription factors (TFs), repressors, co‐activators and chromatin modifying enzymes as well as RNA components like microRNA and other noncoding RNA [2, 3]. Enhancers are primarily *cis*‐acting elements that have been recognized as the pivotal regulators of gene expression and have the ability to activate genes in a distance‐ and orientation‐independent manner. There is overwhelming evidence, based on chromosome conformation capture analysis (3C, 4C and HiC), to suggest that spatial proximity of enhancers and their cognate promoters, achieved via chromatin looping, is critically important [4, 5]. Enhancers have been proposed to act as platforms that can recruit TFs and chromatin remodelling factors [6]. Hence, enhancer‐promoter looping can aid appropriate remodelling of the target promoter and facilitate initiation of transcription and/or release of a poised promoter to an elongating state [7]. However, there is considerable functional and mechanistic diversity in enhancer action [8] and much remains to be understood regarding the molecular basis underlying specificity of enhancer–promoter functional interactions and the mechanisms by which enhancers activate their cognate promoters.

An interesting observation in this context is of the existence of enhancer RNA (eRNA), which is a distinct class of noncoding RNAs transcribed from the enhancer regions. Enhancers of several neuronal‐activity regulated genes were observed to bind CBP and RNA polymerase II (RNAPII) and generate small transcripts termed as eRNA [9]. Similarly, LPS stimulated macrophages exhibited extra‐genic RNAPII binding at known or putative enhancers and their transcription [10]. Discovery of eRNA has provided a new dimension to the research aimed at understanding enhancer‐promoter functional interactions; more so as some other long noncoding RNAs (lncRNAs) have been demonstrated to have a key role in gene regulation exemplified at imprinted loci (*Igf2/H19, Igf2r, Kcnq1*) and *Hox* gene clusters in mammals [11]. Initial studies demonstrated that the eRNAs are transcribed by RNAPII, they are typically nonpolyadenylated and only rarely spliced [9, 12, 13]. They are generally transcribed bidirectionally, are not very stable, are low in abundance and are present in chromatin‐associated fractions during isolation [13]. Transcribed enhancers typically exhibit high levels of H3K4‐me3 [14]. This is unusual as H3K4‐me1 is typically the mark that generally distinguishes enhancers from promoters [15].

Detailed analyses of specific eRNAs *in vivo* have demonstrated tissue specificity in their expression [16, 17, 18]. Further, eRNAs have been demonstrated to be signal responsive and important for stabilizing enhancer‐promoter looping interactions [19, 20] leading to the notion that eRNA have an important role to play in activation of specific promoters by enhancers. Functional eRNAs can potentially be involved in all stages of gene activation by interacting with other RNA and/or proteins. They might contribute to stabilizing of enhancer‐promoter looping interaction, aid enhancer‐dependent generation of promoter chromatin accessibility, RNAPII loading to promoter and release of poised promoter to an elongating stage [21]. While some of these activities would require eRNA transcripts *per se*, merely the process of transcription has also been suggested to be important [21]. Lastly, considering that enhancers act as loading platforms for RNAPII, TFs and chromatin remodelling proteins, in some cases the observed transcripts may also represent transcriptional noise without any functional relevance. Considering the diversity in the functional attributes of mammalian enhancers, the role of eRNAs may be context dependent. It will, therefore, be important to analyse eRNAs produced at different types of enhancer elements.

The enhancer *Eβ* of murine *TCRβ* locus is particularly interesting from a mechanistic viewpoint as it exhibits a combination of looping interactions with target promoters and involvement of some form of tracking to organize the recombination center (RC) critical for VDJ recombination at *TCRβ*. Enhancer *Eβ* is activated in developing thymocytes and regulates transcription as well as VDJ recombination that generates functional genes encoding T‐cell receptor β (*TCRβ*) chains of T‐cell receptors [22]. Previous analysis has established that 560bp region of *Eβ* is critically required for VDJ recombination at TCR*β* locus [23, 24]. Further, this region was sufficient to support VDJ recombination in artificially created miniloci in transgenic mice [25].

*At TCRβ* locus, *Eβ* regulates the chromatin accessibility of 25 kb region encompassing two gene segment clusters, *DJCβ1* and *DJCβ2*, and their promoters, *pDβ1* and *pDβ2*, respectively. Activation of *pDβ1* and *pDβ2* generates germline transcripts from the DJC clusters leading to recruitment of RAG complexes. Enhanced localization of RAG complexes constitutes RC and effects *Dβ*‐to‐*Jβ* recombination. Subsequently, recombined *DJ* regions recombine with *V* segments, which are distantly located but brought to the vicinity of RC by locus contraction. In *Rag1* or *Rag2* knockout mice, neither *Dβ*‐to‐*Jβ* nor *Vβ*‐to‐*DβJβ* recombination takes place and thymocytes are arrested at double negative (DN: CD4^−^CD8^−^) stage in development [26]. In contrast, when both Rag1 and Rag2 are functional, successful VDJ recombination allows development of the thymocytes to double positive (DP: CD4^+^CD8^+^) and eventually single positive (SP: CD4^+^ or CD8^+^) stages. *Eβ* retains its activated status in DP and SP thymocytes and activates the promoter of recombined *TCRβ* gene [27, 28]. The role of *Eβ* is critically important in establishment of RC and VDJ recombination [29, 30, 31]. Chromosome conformation capture analysis [3C‐quantitative PCR (qPCR) and 4C‐ker] demonstrates topological proximity of *Eβ* to upstream *V* segments [32, 33], but its influence on germline transcription of *V* segments and its importance for locus contraction remains moot [34].

Several mechanistic aspects underlying the activity of *Eβ* have been defined. Its looping interaction with target promoters *pDβ1* and *pDβ2* is evidenced by 3C‐qPCR analysis and some form of enhancer tracking also appears to be involved as the chromatin accessibility of a large 25 kb chromatin domain is enhanced as evident by enrichment of acetylated histones [22]. ChIP analysis demonstrated significant binding of RNAPII, CBP and RUNXI at *Eβ* [30, 35]. Looping interactions of *Eβ* with *pDβ1* and *pDβ2* generate a holocomplex and activation of the promoters [30, 35, 36]. Further, 4C‐ker analysis has revealed long‐range interaction between *Eβ* and *MiEk* of *Igk* locus located 26 Mb away [37]. Consistent with the presence of components of transcriptional machinery at *Eβ*, RNA‐seq analysis carried out in developing thymocytes indicated the transcribed nature of *Eβ* [6, 31].

These observations suggest that analysis of eRNA at *Eβ* can provide useful insights in role of eRNA in enhancer‐dependent activation and prompted us to investigate the ongoing transcription at *Eβ* in detail. Our analysis revealed generation of multiple noncoding transcripts at *Eβ* that are polyadenylated and predominantly antisense with respect to the transcripts generated by their cognate promoters.

## Methods

### Experimental mice, *ex vivo* cells and cell lines

*Rag‐1*‐deficient and C57BL/6 mice were procured from Jackson Laboratories, U.S.A., and were bred at animal house facility of NII. All animals were used as approved by Institutional Animal Ethics Committee, NII. *Ex vivo* thymocytes were obtained from thymus of 4‐ to 6‐week‐old mice. Liver cells were obtained from the liver of 2‐ to 3‐day‐old neonates. *Rag‐1*‐deficient thymocyte cell line (P5424) [38] was maintained in RPMI 1640 with 10% FBS.

### Nuclei isolation

Nuclei from P5424 cells were isolated using lysis gradient centrifugation as described earlier [39]. Briefly, 50 million cells were passed through the lysis gradient at 1000 ***g***, 4 °C for 10 min in a swinging bucket rotor. Nuclei were collected from the interface of floating layer and washed once in nuclei isolation buffer (0.25 m Sucrose, 25 mm KCl, 5 mm MgCl_2_ and 60 mm Tris/Cl pH 7.4). Nuclei were collected after spinning at 2000 ***g***, 4 °C for 10 min, and finally resuspended in 50 µL of nuclei suspension buffer (40% v/v Glycerol, 5 mm MgCl_2_, 0.1 mm EDTA and 50 mm Tris/Cl, pH 8.3). Nuclei from *ex vivo* thymocytes of C57BL/6 mice were isolated using sucrose cushion‐based high‐speed ultracentrifugation at 100 000 ***g*** as described earlier [40]. Purified nuclei were taken for further processing as required.

### RNA extraction

RNA from purified nuclei or whole cells was isolated using Trizol reagent (Invitrogen), as per manufacturer's instruction. Isolated RNA was dissolved in DEPC‐treated water and stored at −80 °C till further use.

### Reverse transcription and RT‐qPCR analysis

One microgram of RNA was treated with DNase‐I Amplification grade (Invitrogen) using manufacturer's instruction. DNase‐I‐treated RNA was subsequently used for synthesizing cDNA using Superscript‐III (Invitrogen) by incorporating oligonucleotides as primers [random hexamers, oligo‐dTs or gene‐specific primers (GSPs)] according to manufacturer's protocol. Resultant cDNA was used for end point PCR or qPCR. For strand‐specific reverse transcription quantitative PCR (RT‐qPCR) analysis, biotinylated GSPs were incorporated in a RT reaction. Biotinylated cDNA was purified using Dynabeads MyOne Streptavidin C1 magnetic beads (Invitrogen) using the protocol as previously described by us [41]. Thereafter, 1µl of the enriched biotinylated cDNA was subsequently used in end point PCR or qPCR performed on ABI PRISM SDS7000 or ABI‐QS3. Relative quantitation was performed using genomic DNA (gDNA) for generation of standard curves. Primer sequences are available upon request.

### Rapid amplification of cDNA ends

Five microgram of RNA sample was subjected to 3′ rapid amplification of cDNA ends (RACE) or 5′RACE using RML‐RACE GeneRacer Kit (Invitrogen). For 5′RACE, RNA was dephosphorylated and decapped according to manufacturer's instruction. GeneRacer RNA oligo was ligated to the processed RNA, which was subsequently subjected to RT‐PCR primed with enhancer *Eβ*‐specific primer to detect 5′ transcription start site (TSS) of the capped transcripts. For 3′RACE, cDNA was synthesized from RNA using GeneRacer oligo‐dT primer to detect 3′ transcription end site (TES) of the polyadenylated transcripts. Specific PCR products obtained from RACE were cloned in pGEM‐T Easy vector system (Promega), and ends were determined by Sanger sequencing.

### Analysis of ChIP‐Seq, RNA‐seq and ATAC‐seq datasets

ChIP‐seq datasets for RNAPII, H3K4‐me1 and H3K4‐me3 in developing thymocytes (GSE55635) were previously described [42] and were visualized using Integrated Genome Browser (ver. 9.1.2), http://igb.bioviz.org. RNA‐seq, ATAC‐seq and ChIP‐seq for H3K27‐ac in DN thymocytes were obtained from data series GSE80272 [37] and analysed using Integrative Genomics Viewer (ver. 2.8.9), https://igv.org.

### Coding potential of transcripts

RNA sequence of the putative transcripts based on 5′TSS and 3′TES was used as the input sequence and analysed by the web server of CPC2 algorithm [43] to evaluate their coding potential and presence of ORFs. The coding potential was also evaluated using phylocsf [44, 45] based on the phylogenetic conservation of any protein coding region within the analysed span of *Eβ‐*associated transcripts.

### Statistical analysis

Statistical significance of the difference in relative expression profile of enhancer *Eβ*‐derived transcripts in developing thymocytes and liver cells was estimated using one‐way ANOVA. For strand‐specific RT‐qPCR and subcellular expression analysis, the significance was determined based on *P* values using two‐tailed Student's *t*‐test at 95% confidence interval.

## Results and Discussion

### Active state of enhancer Eβ of murine TCRβ locus is associated with RNAPII binding

Enhancers execute their function in a tissue‐specific manner. At developmentally regulated enhancers, binding of lineage determining pioneering TFs [46] is a critical step that leads to recruitment of diverse chromatin remodelling complexes, co‐activators and other protein complexes [6, 47]. Consequently, an activated state of enhancers is attained that is characterized by its open chromatin configuration with active histone marks such as H3K4‐me1/2/3 and H3K27‐ac. In developing T cells, enhancer *Eβ* of murine *TCRβ* locus has been reported to be activated by the cooperative binding of RUNX1‐ETS protein complexes [27, 48] in DN thymocytes and continues to retain its activated state during subsequent development of thymocytes. It activates the promoters of recombined *TCRβ* genes subsequent to *VDJ* recombination [28] and is crucially required for maintaining the expression of *TCRβ* genes in DP stage of developing thymocytes [27]. Since a large number of enhancers are known to have enhanced occupancy of RNAPII in their active state [9, 10], it raised the question if activated state of *Eβ* also correlates with the occupancy of RNAPII.

**Fig. 1:**
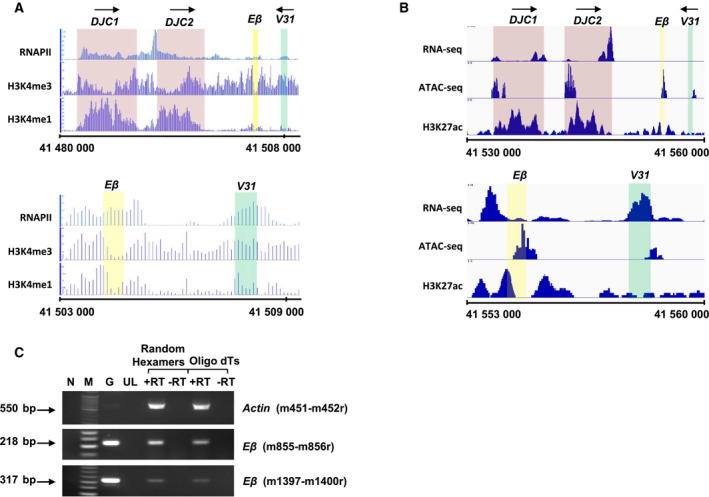
Active transcription at enhancer *Eβ* of *TCRβ* locus in DN thymocytes. Region encompassing the gene segments (*DJCβ1*, *DJCβ2*, *V31*) and enhancer *Eβ* was analysed for several parameters relevant to define transcriptional status. Arrows on the top denote the direction of transcription at specified genic regions. (A) Analysis of ChIP‐seq data showing the occupancy of RNAPII, H3K4‐me1 and H3K4‐me3. Data obtained from published ChIP‐seq dataset GSE55635. Region around enhancer *Eβ* (mm9: Chr 6: 41 480 000–41 509 500) and its magnified view (mm9: Chr 6: 41 503 000–41 509 500) are shown. (B) Analysis of RNA‐seq, ATAC‐seq and ChIP‐seq data showing the enrichment of H3K27‐ac. Data obtained from published data series GSE80272. Region around *Eβ* (mm10: Chr 6: 41 530 000–41 560 000) and its magnified view (mm10: Chr 6: 41 553 000–41 560 000) are shown. (C) PCR amplification of cDNA derived from nuclear RNA of P5425 cells to detect transcripts at *Eβ*. G, gDNA; M, DNA molecular weight marker; N, no template control; UL, unused lanes. +RT and −RT indicate addition of reverse transcriptase during RT‐PCR or its absence, respectively. Primer locations as in Fig. 2.

Analysis of published data from the available ChIP‐seq dataset for RNAPII in developing thymocytes was analysed along with ChIP‐seq datasets for H3K4‐me1 and H3K4‐me3 (GSE55635) [42]. It was observed that enhancer *Eβ* in its activated state as well as ~ 1‐kb region flanking *Eβ* has significant binding of RNAPII in both DN and DP thymocytes (Fig. 1A and Fig. S1). Also, published dataset (GSE80272) consisting of RNA‐seq, ATAC‐seq and ChIP‐seq for H3K27‐ac was analysed from an independent study in DN thymocytes [37]. The analysis revealed that active enhancer *Eβ* was enriched for H3K27‐ac and features an accessible chromatin that yielded low but discernible level of transcription in RNA‐seq analysis (Fig. 1B). Further, FAIRE analysis has also revealed an open and accessible chromatin in the *Eβ* region in DN thymocytes and its association with RNAPII [31].

*Eβ* was associated with H3K4‐me1, H3K27‐ac and H3K4‐me3 although the abundance of these histones at *Eβ* was lower than the genic regions, that is *DJCβ* and *V31* segments (Fig. 1 and Fig. S1). Although many enhancer domains are known to be marked with H3K4‐me1/2 and H3K27‐ac, DNase hypersensitive sites (DHS), along with binding of TFs and cofactor such as CBP/p300 [49], these features are not sufficient to qualify the enhancers to be termed as ‘active’ in a given cell type [49, 50, 51]. Both H3K4‐me1 and H3K27‐ac have been reported to be dispensable for a subset of functional enhancers [49]. They may, however, serve as a landing platform for *trans*‐acting factors such as BRD4 [21, 47]. In fact, *Eβ*, in its inactive state in Pro‐B cells, also exhibits binding of for H3K4‐me1/3, p300 and mediator complex [51] albeit at much lower levels compared to *Eβ* in DN cells [37]. This suggests that open chromatin configuration exists prior to commitment to T‐cell lineage at *Eβ*, as at some other enhancers in their poised or inactive state. In contrast, co‐enrichment or enhanced accumulation of H3K4‐me3 at distal regulatory enhancers was found to correlate well with their activated state in a developmental stage‐specific manner in several T cell‐specific enhancers [42]. Also, the presence of H3K4‐me3 was correlated to active transcription by serine 5‐phosphorylated (Ser‐5P) RNAPII, thereby suggesting that strong enhancers in their active state are enriched for H3K4‐me3 [6, 31, 52]. Therefore, the open state of chromatin at enhancer *Eβ* in developing thymocytes and localization of RNAPII at the enhancer domain is suggestive of ongoing transcription at *Eβ*.

Active enhancers that exhibit RNAPII occupancy have been earlier reported to be transcribed and generate eRNAs that are either polyadenylated or nonpolyadenylated [9, 12, 13, 16, 21]. We first used a *Rag1*‐deficient DN thymocyte cell line (P5424) to address the transcriptional potential of *Eβ*. RT‐PCR analysis using random hexamers and oligo‐dT primers carried out on nuclear RNA of P5424 cells revealed the presence of polyadenylated transcripts emanating from *Eβ* that were more than 300 nucleotides in length (Fig. 1C).

### Bidirectional overlapping transcripts are produced from the active enhancer Eβ in developing thymocytes

**Fig. 2:**
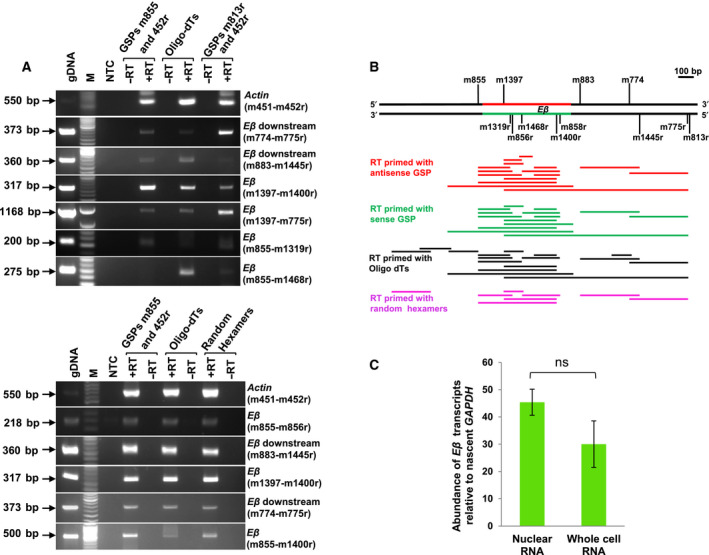
Analysis of transcription at enhancer *Eβ* and its flanking regions by RT‐PCR using random hexamers, oligo‐dT or GSPs to generate cDNA. (A) PCR amplification of cDNA derived from whole cell RNA of *ex vivo* DN thymocytes (top) and nuclear RNA of *ex vivo* total thymocytes (bottom) to detect transcripts. GSP m855 and m813r were used to prime cDNA synthesis for detecting antisense and sense transcripts from *Eβ*, respectively. GSP m452r for detecting *β‐Actin* mature mRNA was incorporated in RT to provide a positive control. Primers for PCR amplicons tested are mentioned. (B) Schematic representation of several PCR amplicons used for the RT‐PCR analysis in *ex vivo* developing thymocytes. Red and green solid lines represent sense and antisense strand of *Eβ* span, respectively. (C) Relative abundance of *Eβ* transcripts in nuclear and whole cell RNA of *ex vivo* total thymocytes. Data depicts mean ± standard deviation of three experiments. Two‐tailed Student's *t*‐test was used to determine statistical significance. NTC, no template control, ‐RT, no reverse transcriptase added in RT‐PCR. M and UL represent DNA molecular weight marker and unused lanes, respectively.

After confirming the transcriptional potential of enhancer *Eβ* by the preliminary RT‐PCR analysis in P5424 cells, the directionality of eRNA transcription was determined in *ex vivo* thymocytes, since transcription at the enhancers can occur both unidirectionally or bidirectionally. Consistent with observations in P5424 cells, polyadenylated transcripts from *Eβ* and the flanking downstream region were also detected in RNA isolated from DN thymocytes (derived from *Rag1^−/−^* mice). Furthermore, transcripts as long 1.1 kb were observed in both sense and antisense orientation (Fig. 2A, top panel) as evidenced by GSPs used to prime reverse transcription. These bidirectional transcripts completely overlapped each other, thereby representing a sense–antisense RNA pair. Such co‐expressing overlapping transcripts have been reported at several other loci and have been termed as natural antisense transcripts (NATs) [3]. However, no amplification was observed by PCR at the upstream region flanking *Eβ* (m855‐m1468r) for antisense transcripts. This might be due to low level of antisense transcripts at the examined upstream region.

Next, similar RT‐PCR analysis was performed on nuclear RNA of total thymocytes isolated from C57BL/6 mice and reasonably long polyadenylated transcripts in both antisense (Fig. 2A, bottom panel) and sense orientation (data not shown) were detected from *Eβ* and the flanking downstream region. Further, transcription was also observed at the upstream region flanking *Eβ* as summarized in Fig. 2B. These observations indicate that active enhancer *Eβ* is transcribed bidirectionally to produce polyadenylated transcripts and extend the observation of low level of transcription at *Eβ* based on RNA‐seq analysis in both DN and DP thymocytes [6, 31]. Since *Eβ* remains active in DP and SP thymocytes [27], it was not surprising that the transcripts continued to be expressed in the total thymocytes as inferred by our RT‐PCR analysis.

It was intriguing to note that the transcription from *Eβ* was found to be overlapping and convergent. It is likely that the observed transcription from each strand of *Eβ* occurs in a mutually exclusive manner and that only one strand at a time is transcribed in an individual cell. Recently, it has been realized that the perceived bidirectional transcription from the enhancers occurs in a mutually exclusive manner and eRNAs are transcribed unidirectionally in a given cell [53].

*Eβ* transcripts were evident in nuclear RNA as well as whole cell RNA (Fig. 2C). The detected transcripts at *Eβ* were found to be localized in the nucleus, and the abundance of transcripts was not significantly reduced after stripping off the cytoplasm. Whether they remain confined at the site of synthesis associated with chromatin or additionally accumulate at other regions is not currently known. Our observations are consistent with previous reports where eRNAs have been found to be predominantly enriched in the nuclear compartment [10, 53, 54]. While the transcripts were largely localized in the nuclei, they were easily detectable in the whole cell RNA.

### Transcripts at Eβ have multiple 5′TSS and 3′TES

**Fig. 3:**
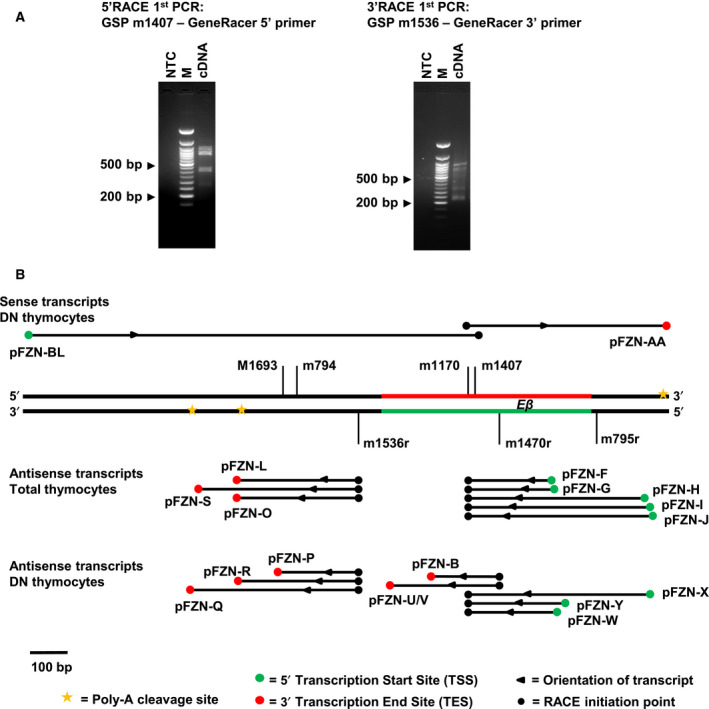
Detection of 5′TSS and 3′TES for *Eβ* transcripts in *ex vivo* DN and total thymocytes. (A) Typical amplifications observed during 5′ and 3′ RACE. *Left:* 1^st^ PCR amplification of 5′RACE ready cDNA generated by using GSPm1170 to detect antisense transcripts. Right: 1^st^ PCR amplification of 3′RACE ready cDNA generated by using GeneRacer oligo‐dT primer to detect antisense transcripts. (B) Location of various 5′TSS and 3′TES of the sense and antisense transcripts as detected after sequencing of cloned RACE products in DN and total thymocytes. Red and green solid lines represent sense and antisense strand of *Eβ* span, respectively. Primers used in RACE analysis are marked along the span of *Eβ* and the flanking regions in the map. M, 50 bp DNA molecular weight marker; NTC, no template control.

To characterize the transcripts at *Eβ*, RACE was performed for identifying their TSSs (5′TSS) and TESs (3′TES). Our analysis of the antisense transcripts in the nuclear RNA of total thymocytes revealed multiple 5′TSS both within and downstream to *Eβ* (Fig. 3A,B and Table 1). These 5′TSS were broadly distributed in a region of 300 nucleotides and transcripts were found to be capped as the assay was designed for detecting specifically the capped transcripts. At their 3′ ends, the transcripts were confirmed to be polyadenylated by the sequencing of 3′RACE products and multiple 3′TES were mapped upstream *Eβ* in total thymocytes (Fig. 3A,B and Table 1).

**Table 1:** Location of the 5′TSS and 3′TES of antisense transcripts in total thymocytes. Precise start and end sites of the transcripts are mentioned with their respective mm10 coordinates as determined by sequencing of cloned 5′RACE and 3′RACE products for antisense transcripts in *ex vivo* total thymocytes.

Clone ID	Start/end	Location	mm10 coordinates of 5′TSS on chr 6	mm10 coordinates of 3′TES on chr 6
pFZN‐F	5′ TSS	Within *Eβ*	41 554 626	NA
pFZN‐G	5′ TSS	Within *Eβ*	41 554 632	NA
pFZN‐H	5′ TSS	Downstream *Eβ*	41 554 893	NA
pFZN‐I	5′ TSS^a^	Downstream *Eβ*	41 554 909	NA
pFZN‐J	5′ TSS	Downstream *Eβ*	41 554 912	NA
pFZN‐L	3′ TES^a^	Upstream *Eβ*	NA	41 553 795
pFZN‐O	3′ TES	Upstream *Eβ*	NA	41 553 790
pFZN‐S	3′ TES	Upstream *Eβ*	NA	41 553 693

^a^ Represents sites that were found to be identical in DN thymocytes.

Similarly, the antisense transcription in DN thymocytes was also characterized by multiple 5′TSS and 3′TES, and the transcripts were found to be capped and polyadenylated in 5′ and 3′ RACE, respectively (Fig. 3A,B and Table 2). Distinctly from observations made on total thymocytes, 3′TES of antisense transcripts in DN thymocytes was found both within and upstream *Eβ*. While a subset of 3′TES of the transcripts were detected in close vicinity of the canonical poly‐A cleavage motifs in the underlying sequence, as identified bioinformatically, additional 3′TES were also detected. Furthermore, cDNA synthesized with the primer (m1693) located in the vicinity of 3′TES identified with pFZN‐P gave a PCR amplicon (m794‐m795r) that covered the complete span from 3′TES pFZN‐P to 5′TSS pFZN‐X (Fig. S2). This suggests that at least 3′TES identified with pFZN‐P is in continuation with all the identified 5′TSS in DN thymocytes and an antisense transcript from pFZN‐X to pFZN‐P, termed as *as‐RNA‐xp*, can have a size of 993 bases. Since the 3′TES detected within *Eβ* were also covered with the PCR amplicon m794‐m795r, it was not possible to further connect them with any specific 5′TSS. These sites can together represent several small transcripts ranging in size from ~ 300 to 700 bases. While 5′ and 3′ RACE revealed the existence of several transcripts, the possibility of additional transcripts cannot be ruled out that might be missed during RACE especially if their abundance was low.

**Table 2:** Location of the 5′TSS and 3′TES of antisense transcripts in DN thymocytes. Start and end sites of the transcripts are mentioned with their respective mm10 coordinates as determined by sequencing of cloned 5′RACE and 3′RACE products for antisense transcripts in *ex vivo* DN thymocytes.

Clone ID	Start/end	Location	mm10 coordinates of 5′TSS on chr 6	mm10 coordinates of 3′TES on chr 6
pFZN‐W	5′ TSS	Within *Eβ*	41 554 637	NA
pFZN‐Y	5′ TSS	Within *Eβ*	41 554 673	NA
pFZN‐X	5′ TSS^a^	Downstream *Eβ*	41 554 909	NA
pFZN‐P	3′ TES	Upstream *Eβ*	NA	41 553 916
pFZN‐Q	3′ TES	Upstream *Eβ*	NA	41 553672
pFZN‐R	3′ TES^a^	Upstream *Eβ*	NA	41553 795
pFZN‐B, C & D	3′ TES	Within *Eβ*	NA	41 554 325
pFZN‐U & V	3′ TES	Within *Eβ*	NA	41 554 214

^a^ Represents sites that were found to be identical in total thymocytes.

For sense transcripts in DN thymocytes, a single 5′TSS (pFZN‐BL) and a 3′TES (pFZN‐AA) pertaining to capped polyadenylated RNA were mapped in the upstream and downstream region flanking *Eβ*, respectively (Fig. 3B and Table 3). Though several amplicons were obtained in 5′RACE PCR, only one amplicon (pFZN‐BL) was found to be specific to the *Eβ* upstream region in our RACE analysis for sense transcripts. Together, 5′TSS pFZN‐BL and 3′TES pFZN‐AA represent a 1.7 kb sense transcript, termed as *s‐RNA‐blaa*, in DN thymocytes. Similar to antisense transcripts, some 5′TSS and 3′TES also may have remained unidentified in the RACE analysis for sense transcripts.

**Table 3:** Location of the 5′TSS and 3′TES of sense transcripts in DN thymocytes. Start and end sites of the transcripts are mentioned with their respective mm10 coordinates as determined by the sequencing of cloned 5′RACE and 3′RACE products for sense transcripts in *ex vivo* DN thymocytes.

Clone ID	Start/end	Location	mm10 coordinates of 5′TSS	mm10 coordinates of 3′TES
pFZN‐AA	3′TES	Downstream *Eβ*	NA	41 554 955
pFZN‐BL	5′ TSS	Upstream *Eβ*	41 553 229	NA

*Eβ*, as defined by deletion analysis [23, 24], is located about 6 kb downstream to 3′TES of *DJCβ2* transcription unit and 3 kb upstream to TES of gene segment *V31,* which is transcribed in antisense orientation. Potentially, read‐through transcription from these transcription units can be manifested as transcription at the enhancer *Eβ*. However, RNAPII enrichment peak at *Eβ* was well separated from the peaks at the neighbouring transcription units, suggesting that the detected transcripts at *Eβ* were not a consequence of read‐through transcription. This contention was also supported by the detection of transcription start site by 5′RACE within and in close proximity of *Eβ*.

Altogether, bidirectional transcripts at enhancer *Eβ* were found to be completely overlapping and were characterized by substantial heterogeneity at both the ends. Further these transcripts were capped and polyadenylated at both strands and apparently were co‐expressed in both the orientations. In several studies, DHS regions mapping enhancers have been found to be enriched in CAGE tag libraries [12, 53] where transcriptional activity was associated with ser‐5P RNAPII [6, 31], a form that helps to recruit cap‐binding complex [6, 10, 54]. Furthermore, 50% of the enhancer regions have been reported to have widely dispersed and overlapping CAGE tags, suggesting that enhancers were transcribed into capped transcripts and display heterogeneity at their 5′TSS [10, 50]. The eRNAs have been predominantly reported as nonpolyadenylated and bidirectional transcripts [6, 9, 12], whereas polyadenylated transcripts at other distal regulatory regions, including few enhancers, have always been reported to transcribe in a unidirectional fashion [6, 10, 16]. Our results, however, revealed existence of polyadenylated bidirectional transcripts. These bidirectional transcripts at *Eβ*, as at many other enhancer regions, may actually represent a pool of transcripts expressed from both the strands in a given cell population, which are otherwise transcribed in a unidirectional fashion from a given allele at single cell level. RNA‐FISH analysis at several bidirectionally transcribed enhancers has indeed revealed that both the strands are only rarely co‐expressed from the same allele in a cell [53, 55].

### Transcripts from enhancer Eβ are expressed specifically in developing T cells

**Fig. 4:**
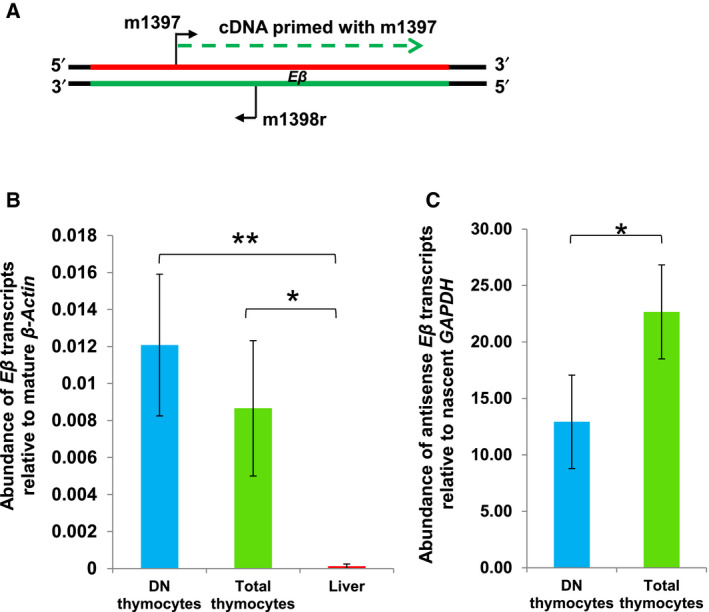
Abundance of transcripts detected at *Eβ* by RT‐qPCR in the active and inactive state of *Eβ*. (A) Schematic representation of primer positions for cDNA synthesis and subsequent qPCR amplification. (B) Expression of transcripts generated at *Eβ* in DN thymocytes, total thymocytes and neonatal liver relative to mature *β‐Actin* transcripts. One‐way ANOVA was used to determine statistical significance. (C) Comparison of antisense transcripts at *Eβ* in DN and total thymocytes relative to nascent *GAPDH* transcript. Statistical significance was determined by two‐tailed Student's *t‐*test. Data depict mean ± standard deviation of three biological replicates (*P < 0.05 and **P < 0.01).

Being regulatory elements, enhancers are known to exhibit highly restrictive expression of transcripts at low levels in specific cell types in correlation with their active state [6, 12]. Also, many eRNAs have been reported to be compartmentalized in the nuclei, wherein they are associated with the chromatin to exert their regulatory functions [10, 16, 53]. As stable transcripts were detected at enhancer *Eβ*, we examined their levels in cells where *Eβ* is in active state, that is DN thymocytes and total thymocytes and compared with levels in cells where *Eβ* is inactive, that is cells of neonatal liver. Utilizing RT‐qPCR analysis, we observed that their expression is restricted to the thymocytes. In liver, the expression was negligible compared to the thymocytes (Fig. 4A,B).

Further, the relative levels of these transcripts were found to be nearly 100‐fold less than the mature *β‐Actin* transcripts in developing thymocytes (Fig. 4B) and consistent with previous reports demonstrating discernible but relatively low accumulation of RNAPII at the enhancer *Eβ* [31, 42]. We observed the levels of *Eβ* transcripts to be higher in DN thymocytes than total thymocytes but both of them exhibited a large variation in the estimations. The variations in levels may be partly attributed to low level of transcription. Alternatively, variation in quantitation can also arise due to ‘background priming’, a phenomenon where cDNA can be synthesized from both the strands due to self‐priming by RNA itself [56]. Background priming can interfere in accurate quantitative analysis of expression especially in context of overlapping transcripts.

To overcome the problem of background priming in RT‐PCR, we employed biotinylated primers for strand‐specific RT‐qPCR analysis and used stringent tests to ensure accurate detection of transcripts in a strand‐specific manner, using a protocol previously reported by us [41]. We first analysed the antisense transcripts using the strand‐specific RT‐qPCR analysis. Antisense transcripts from *Eβ* were found to be approximately 12.5‐fold more abundant than nascent *GAPDH* transcripts in whole‐cell RNA of DN thymocytes. In total thymocytes, these transcripts exhibited about 22.5‐fold higher expression than nascent *GAPDH* (Fig. 4A,C). In contrast, multiple attempts to quantitate sense transcripts using strand‐specific RT‐qPCR were unsuccessful in DN as well as total thymocytes. Clearly, although transcribed and detectable during RACE, the sense strand‐specific transcripts are much less abundant than antisense transcripts. Differential expression of bidirectional transcripts emanating from enhancers has also been reported at *Arc* locus [57] and at the enhancer of KLK3 gene [58]. Favoured transcription from a specific strand has been linked with the directional preference of RNAPII to initiate or continue transcription elongation [54].

### Transcripts detected at enhancer Eβ lack coding potential

The coding potential of a subset of transcripts detected at *Eβ* was assessed bioinformatically. RNA sequences of several sense and antisense transcripts from a given 5′TSS to 3′TES were analysed using CPC2 algorithm [43]. All the transcripts detected at *Eβ* were found to have coding probability < 0.1 and were classified as noncoding. The transcripts were also analysed for coding any putative small peptides and were found to have low scores for the same. The scores for small peptide coding probability were found to be comparable to the well‐established noncoding RNAs such as *Arc eRNA*, *^DRR^eRNA* and *HOTAIR* (Table 4). Altogether, the transcription from *Eβ* seems to be noncoding in nature and has very low probability for translating into small peptides. As a complementary approach, the noncoding nature of *Eβ* associated transcripts was further ascertained using PhyloCSF [44, 45], which aims to detect phylogenetically conserved protein coding regions in a given sequence. PhyloCSF analysis for a sequence that encompass the span of sense and antisense transcripts detected at *Eβ* yielded negative scores in all the six frames (Fig. S3), thereby substantiating the noncoding nature of these transcripts.

**Table 4:** Coding potential of various transcripts in the mouse genome as predicted by CPC2 algorithm. Coding probability has been rounded off to 5th decimal place. AA and nt refer to amino acids and nucleotides, respectively.

Transcript	5′TSS & 3′TES	Length of the transcript (nt)	Length of the putative ORF	Coding probability
*s‐RNA‐blaa*	pFZN‐BL and pFZN‐AA	1727	93 AA	0.09568
*as‐RNA‐xp*	pFZN‐X and pFZN‐P	994	52 AA	0.01838
*as‐RNA‐yp*	pFZN‐Y and pFZN‐P	758	52 AA	0.01879
*as‐RNA‐wp*	pFZN‐W and pFZN‐P	722	52 AA	0.01834
*as‐RNA‐xq*	pFZN‐X and pFZN‐Q	1238	52 AA	0.01897
*as‐RNA‐xr*	pFZN‐X and pFZN‐R	1116	52 AA	0.01900
*Arc eRNA*	–	857	108 AA	0.44724
*Cga eRNA* (forward)	–	1506	57 AA	0.05143
*Cga eRNA* (opposite)	–	1050	36 AA	0.01273
*^DRR^eRNA*	–	2126	63 AA	0.02805
*HOTAIR*	–	2006	35 AA	0.00867
*HOTTIP*	–	2879	77 AA	0.05036
*NEAT1*	–	3190	80 AA	0.04823
*RUNX1* mRNA	–	1398	466 AA	1
*GAPDH* mRNA	–	1002	333 AA	0.54073

## Conclusions

This study was undertaken to gain deeper insight into the nature of the noncoding transcription at enhancer *Eβ*. In our analyses, transcripts from enhancer *Eβ* were typically longer than 200 nucleotides, capped, polyadenylated and predominantly in antisense orientation. They were expressed specifically in developing thymocytes where *Eβ* is active. Further, they were largely nuclear in localization and noncoding. Several of these characteristics suggest that the transcripts can be classified as eRNA. However, many of these features are shared between eRNA and lncRNA making a strict classification between these two types of noncoding transcripts difficult [59, 60, 61]. Further, *cis*‐regulatory elements such as enhancers and promoters exhibit several commonalities and can switch roles in a context‐dependent manner [52]. Intragenic enhancers have been demonstrated to act as alternative promoters that were transcribed into polyadenylated lncRNAs [62]. Also, transcribed enhancers were found to exhibit weak promoter activity in their transcribed orientation [63]. Conversely, the alternative promoters undergoing bidirectional transcription were demonstrated to possess enhancer activity. Thus, a gradient exists between enhancers and promoters in their transcribed state as well as ability to regulate transcription distally. Considering the ambiguity between the inherent features of a subset of enhancers and promoters, it is possible that the transcripts at *Eβ* might constitute lncRNAs. Delineation of the functional roles of *Eβ*‐associated RNA may be helpful in their classification as eRNA or lncRNA. It has been considered that eRNA are shorter and act locally, while lncRNA are longer, stable and can act in *trans*. However, the ambiguities remain even if functionality is considered as some eRNA have been reported to function as lncRNA, while conversely, some lncRNA have been observed to be associated with an enhancer. More analyses of several noncoding RNA are required to clearly define the various categories of noncoding RNA [59].

Although majority of eRNA are capped but not polyadenylated, we observed that the transcripts at *Eβ* were capped as well as polyadenylated. Most of the nonpolyadenylated eRNAs are unstable with a short half‐life of ~ 7 min [10, 57] and thus lack the stability of polyadenylated mRNAs [53, 54]. The cap‐binding complex at 5′end of eRNAs has been shown to facilitate the recruitment of exosomal machinery leading to their rapid turnover [12, 50], Interestingly, the susceptibility of eRNAs to exosomal machinery has been reported to have inverse correlation with their length [21]. Longer eRNAs with 3′ polyadenylated tails tend to exhibit higher stability than their nonpolyadenylated shorter counterparts [16, 21, 54]. Since the polyadenylated transcripts detected at *Eβ* were significantly longer (mean length > 1 kb) than the nonpolyadenylated eRNAs detected in FANTOM5 CAGE libraries (median length of 346 nucleotides) [12], it can be speculated that they are less susceptible to exosomal machinery based turnover. Thus, polyadenylation may confer a higher degree of stability to the eRNAs. Consistent with the reported observations [21], the 2 kb long *^DRR^eRNA*, expressed in myotubules, exemplifies a fairly stable polyadenylated noncoding transcript with a half‐life of ~ 30 min and acts in *trans* to regulate the expression of *Myogenin* [16]. Despite being polyadenylated, the abundance of *Eβ* based transcripts was low. Whether the abundance was low due to lower degree of transcript initiation or instability of the transcripts remains a question. Also, additional mechanisms that regulate steady state levels of the transcripts cannot be ruled out.

Noncoding transcription can exert its function in diverse ways. Recently, it has been proposed that eRNAs can orchestrate higher order chromatin organization by interacting with multivalent protein complexes, leading to liquid‐liquid phase separation of the supra‐molecular assemblies along with chromatin as a means to confine their biochemical activity in a membrane‐less compartment [64]. Therefore, it can be hypothesized that the observed transcripts at *Eβ* can bind specific proteins that are crucial to stabilize its interaction with the upstream promoters and/or have a role in their subsequent activation. Since the association of eRNAs with chromatin modifying factors can influence both their binding and activity [47, 65], they can collaborate to maintain the RC in its highly active state at *TCRβ* locus.

Transcription of 3′RR super‐enhancer of *Igh* locus was recently reported to be crucial to facilitate its transition from activated to truly functional state necessary for class switch recombination (CSR) [66]. In the absence of eRNA, consequent to Spt5 depletion, the deposition of H3K27‐ac, mediator and cohesion complexes was unaffected but interaction of 3′RR with target promoter (*Ighg1*) was significantly reduced leading to loss of germline transcription essential for CSR. 3′RR transcription was required for establishment, albeit not maintenance, of long‐range interactions between 3′RR‐*Igh* promoters. Importantly, regulation of multiple enhancer–gene pairs was differentially affected by Spt5 depletion in this study and emphasized the context dependence of the eRNA in terms of its role as well as mechanism of action – an aspect that will continue to stimulate further investigations to gain useful insights in the realm of gene regulation and other nuclear processes.

Additional roles of *Eβ*‐associated transcripts cannot be excluded as relatively long and polyadenylated stable eRNA have been reported to have the ability to act in *trans* [16, 67]. The characterization of transcripts at *Eβ* will provide an opportunity to dissect the role of eRNAs in regulating enhancer activity.

## Conflict of interest

The authors declare no conflict of interest.

## Author contributions

MS conceived the study. FU and MS designed by the experiments and FU performed the experiments. FU and MS analysed the data. FU and MS prepared the manuscript.

## Supporting information


**Fig. S1.** Analysis of ChIP‐seq data showing the occupancy of RNAPII, H3K4‐me1 and H3K4‐me3 at murine *TCRβ* locus in DP thymocytes.
**Fig. S2.** Assessment of the span of antisense transcripts in *ex vivo* DN thymocytes by strand‐specific RT‐PCR using biotinylated cDNA.
**Fig. S3.** PhyloCSF analysis of the region encompassing sense and antisense transcripts detected at active enhancer *Eβ*.

## Data Availability

Data will be available from the corresponding author upon reasonable request.
