# Supplementary material for: Characterization of transcripts emanating from enhancer Eβ of the murine TCRβ locus
Source: FEBS Open Bio. 2021 Mar 16;11(4):1014–28. doi: 10.1002/2211-5463.13079 (PMC8016127; doi:10.1002/2211-5463.13079)
Supplement: Supplementary file 1 — Fig. S1. Analysis of ChIP‐seq data showing the occupancy of RNAPII, H3K4‐me1 and H3K4‐me3 at murine TCRβ locus in DP thymocytes. Fig. S2. Assessment of the span of antisense transcripts in ex vivo DN thymocytes by strand‐specific RT‐PCR using biotinylated cDNA. Fig. S3. PhyloCSF analysis of the region encompassing sense and antisense transcripts detected at active enhancer Eβ. [file FEB4-11-1014-s001.pdf]

Supplementary Figure 1

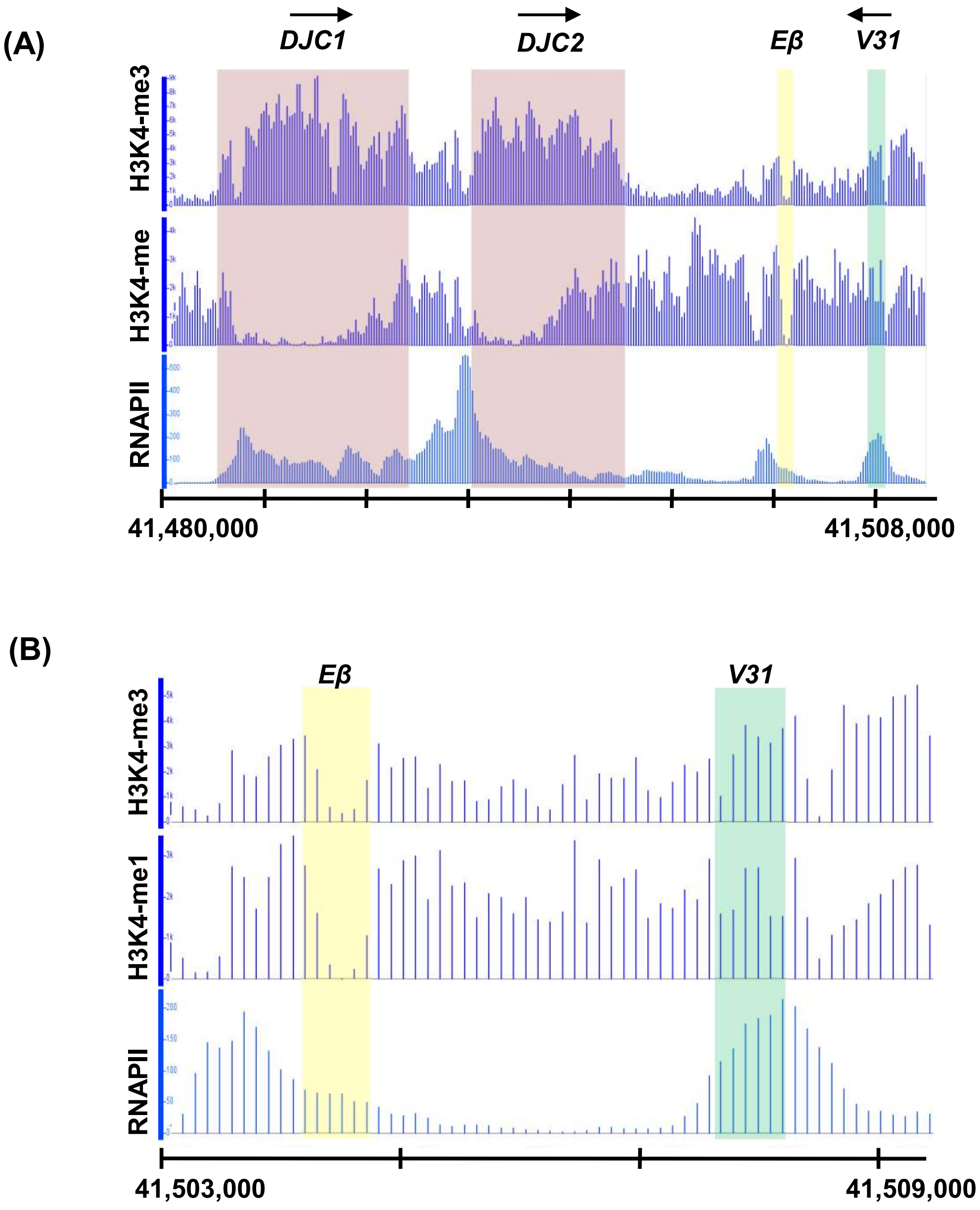

Supplementary Figure 1. Analysis of ChIP-seq data showing the occupancy of RNAPII, H3K4-me1 and H3K4-me3 at murine *TCRβ* locus in DP thymocytes. Data obtained from published ChIP-seq dataset GSE55635. **(A)** Chromatin profile of ~29 kb region encompassing the gene segments (*DJCβ1* and *DJCβ2*), enhancer *Eβ* and *V31* (mm9: Chr 6: 41480000-41509500). Arrows on the top denote the direction of transcription at specified genic regions. **(B)** Magnified view of the chromatin profile around enhancer *Eβ* (mm9: Chr 6: 41,503,000-41,509,500).

Supplementary Figure 2

(A)

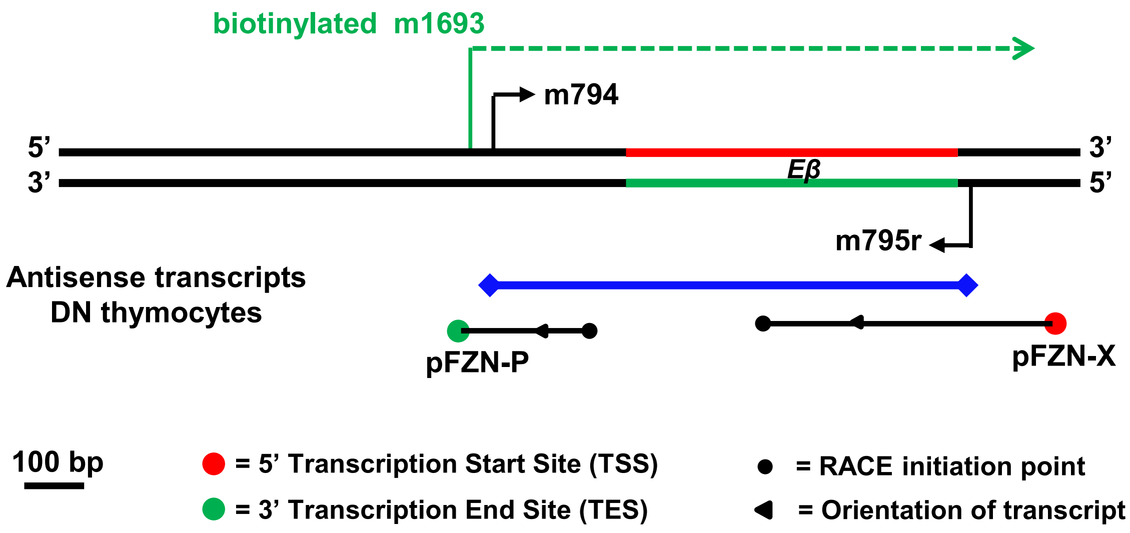

(B)

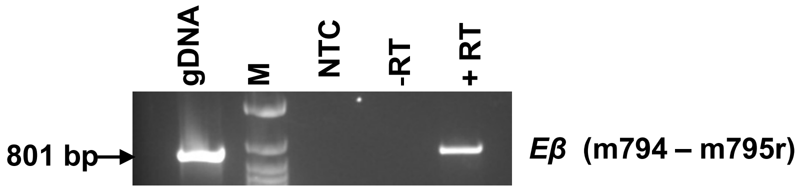

Supplementary Figure 2. Assessment of the span of antisense transcripts in *ex-vivo* DN thymocytes by strand specific RT-PCR using biotinylated cDNA. **(A)** Position of primers used for cDNA synthesis and PCR. Red and green solid lines represent sense and antisense strand of *Eβ* span, respectively. Dashed green line and blue line refer to the biotinylated cDNA synthesized and the PCR amplicon detected, respectively. pFZN-P and pFZN-X represent a 3'TES and 5'TSS as detected by 3' RACE and 5'RACE for antisense transcripts in DN thymocytes (Figure 3). **(B)** PCR amplification of enriched biotinylated cDNA synthesized using GSP m1693 to detect antisense transcripts. Amplicon m794-m795 was detected by PCR. NTC, no template control; M, DNA molecular weight marker.

Supplementary Figure 3

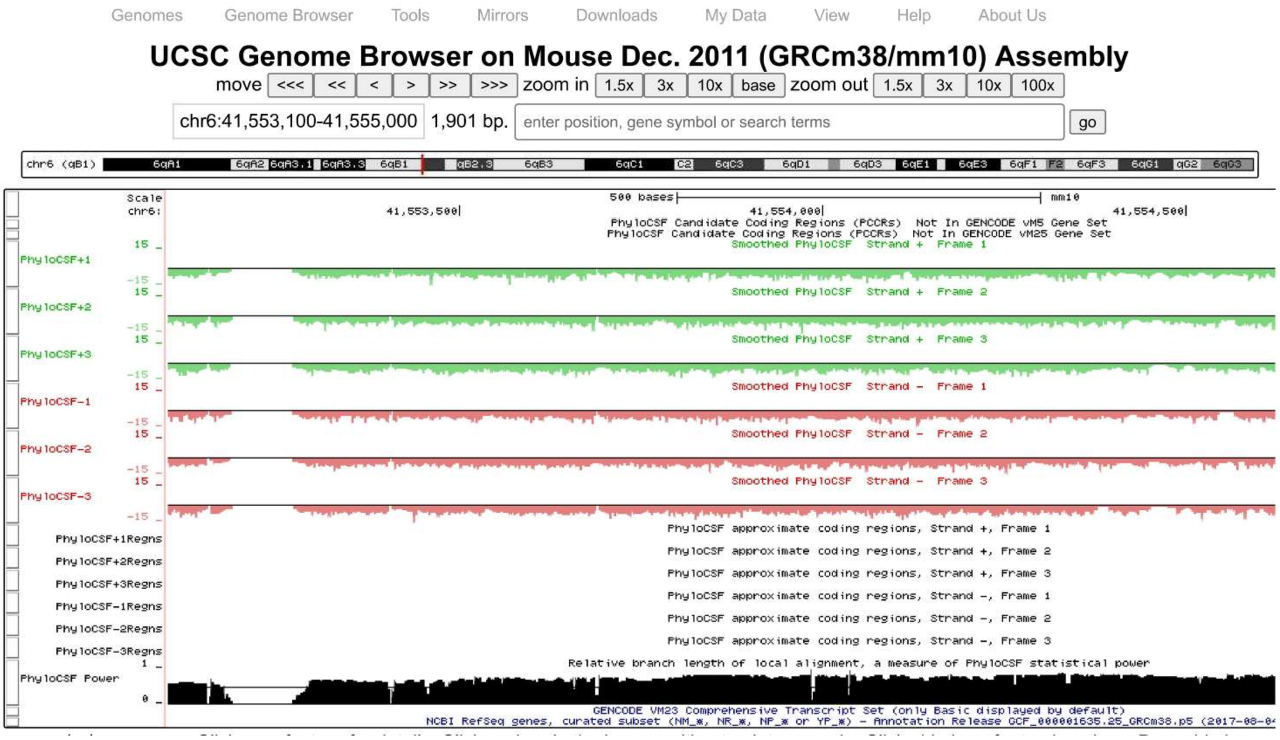

Supplementary Figure 3. PhyloCSF analysis of the region encompassing sense and antisense transcripts detected at active enhancer *Eβ*. The analysis was carried out on the PhyloCSF track hub of UCSC Genome Brower using Chromosomal coordinates mm10:chr6:41,553,100-41,555,000.
